# 3D Model of Heat Flow during Diffusional Phase Transformations

**DOI:** 10.3390/ma16134865

**Published:** 2023-07-06

**Authors:** Łukasz Łach, Dmytro Svyetlichnyy

**Affiliations:** AGH University of Krakow, Faculty of Metals Engineering and Industrial Computer Science, al. Mickiewicza 30, 30-059 Krakow, Poland; svetlich@metal.agh.edu.pl

**Keywords:** phase transformation, heat flow, enthalpy, frontal cellular automata, lattice Boltzmann method, CUDA

## Abstract

The structure of metallic materials has a significant impact on their properties. One of the most popular methods to form the properties of metal alloys is heat treatment, which uses thermally activated transformations that take place in metals to achieve the required mechanical or physicochemical properties. The phase transformation in steel results from the fact that one state becomes less durable than the other due to a change in conditions, for example, temperature. Phase transformations are an extensive field of research that is developing very dynamically both in the sphere of experimental and model research. The objective of this paper is the development of a 3D heat flow model to model heat transfer during diffusional phase transformations in carbon steels. This model considers the two main factors that influence the transformation: the temperature and the enthalpy of transformation. The proposed model is based on the lattice Boltzmann method (LBM) and uses CUDA parallel computations. The developed heat flow model is directly related to the microstructure evolution model, which is based on frontal cellular automata (FCA). This paper briefly presents information on the FCA, LBM, CUDA, and diffusional phase transformation in carbon steels. The structures of the 3D model of heat flow and their connection with the microstructure evolution model as well as the algorithm for simulation of heat transfer with consideration of the enthalpy of transformation are shown. Examples of simulation results of the growth of the new phase that are determined by the overheating/overcooling and different model parameters in the selected planes of the 3D calculation domain are also presented.

## 1. Introduction

Modern technological progress increases the requirements for the materials used. The development of materials science, materials engineering, and metallurgy is heading toward the production of materials with properties that meet the expectations of more and more demanding users. One of the methods which can improve the properties of steel products and, consequently, the quality of finished products, is heat treatment [1]. The current market requires thorough analysis and control of the condition of materials that are elements in the design and production processes of the final product.

Phase transformation processes in metals and alloys are one of the basic factors through which the structure and properties of metallic construction materials can be influenced [2]. The task of this field of technical knowledge is to determine the influence of changes in external conditions on the structure of metals and alloys, and to determine the relationship between the composition and structure of alloys and their properties. Understanding these relationships requires knowledge of elementary processes that occur in the material under the influence of temperature changes and other external factors. Knowledge of the kinetics of transformations allows researchers to control their course in such a way as to obtain the assumed desired result.

Thermodynamic parameters distinguish the first- and second-order transitions. Many metallurgical phase transformations demonstrate features typical for first-order transition, such as type of nucleation and grain growth, strain energy generation, and a sharp interface between parent and new phases [3]. The second-order transformation is a transformation in which a crystal structure undergoes a continuous change and the first derivatives of the Gibbs energies (or chemical potentials) are continuous but the second derivatives with respect to temperature and pressure (i.e., heat capacity, thermal expansion, compressibility) are discontinuous [4].

In metallurgy, the classification related to the transformation mechanism is of great importance, according to which the following groups of transformations are distinguished [5]:

- Diffusion—the course of which is related to mass transport (over short or long distances), e.g., eutectoid transformation, separation of components from supersaturated solid solutions.
- Diffusionless—not requiring mass transport, e.g., martensitic transformation.

**Figure 1:**
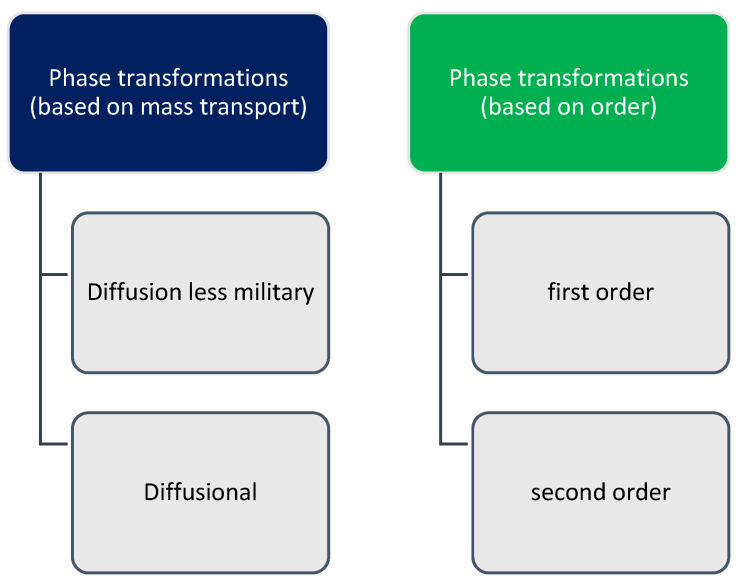
Classification of phase transformations.

Figure 1 shows the classification of phase transformations based on mass transport and phase transitions based on order.

Over the years, various experimental methods for studying phase transformations have been developed. In 1930, Davenport and Bain used the isothermal transformation as a technique to study phase transformations as a function of time and temperature [6]. Illustrating the phase changes occurring depending on the temperature of the solution and the elapsed time during the isothermal transformation is possible using the time–temperature–transformation chart 𝐶𝑇𝑃𝑖. In this diagram, the occurrence of characteristic C-type curves separating the areas of occurrence of individual austenite decomposition products is observed. The 𝐶𝑇𝑃𝑖 chart became the basis for much further research in this area. The historical development of the understanding of phase transformations in ferrous alloys was presented in the work of Hackenberg [7]. Among recent works related to experimental studies of phase transformations, the following can be mentioned: the theoretical and experimental study of phase transformation and twinning behavior in metastable high-entropy alloys [8], dilatometric study of phase transformations under conditions of recrystallized and non-recrystallized austenite in 3Mn1.5Al steel [9], solid–solid phase transformations and their kinetics in Ti-Al-Nb alloys [10], and the study of phase transformation and mechanical properties of ultrahigh strength steels under continuous cooling conditions [11]. An area that is also heavily explored is diffusional phase transformations, and, in recent years; for example, Kumar et al. have studied the diffusional transformations using in situ cooling and heating techniques in a scanning electron microscope [12], while Mueller et al. [13] have studied diffusional and partitionless ferrite-to-austenite phase transformations during intercritical annealing of medium-Mn steels.

During phase transformations of metals and alloys, latent heat of transformation is released or absorbed; therefore, the curves of temperature change as a function of time show stops or inflection points caused by deviations from the normal course of the heating or cooling curve [14]. From these curves, it is possible to determine the temperature or range of temperatures at which a given transformation occurs. In the simplest case of thermal analysis, the test specimen is heated or cooled in a furnace that has a zone of uniform temperature distribution larger than the length of the sample. The rate of heating and cooling should be constant and, in the case of steel, low enough for phase changes to take place in conditions close to equilibrium.

The topics related to latent heat have been and continue to be the subject of many model studies carried out in various areas. Recently, Inkeri et al. realized the numerical modeling of latent heat thermal energy storage integrated with the heat pump for domestic hot water production [15]. Proell et al. described phase change and latent heat models for the simulation of metal powder bed fusion additive manufacturing processes on the macroscale level [16]. Scoggin et al. modelled the latent heats of crystallization and fusion in phase change materials with a unified latent heat of phase change, ensuring energy conservation by coupling the heat of phase change with amorphous and crystalline specific heats [17]. The latent heat release in 3D phase field simulations of dendritic crystal growth was studied by Strachota et al. [18].

Different phenomena and processes in materials can be modeled using a wide range of different numerical methods. The requirements and assumptions that underline the process in question can determine the appropriate modeling method. This also applies to the modeling of phase transformations in materials, where different methods are used. Recently, a study of the kinetics of peritectic phase transformation with elastic effect in the Fe–C system, based on quantitative phase-field modeling, was realized by Parida et al. [19]. Application of phase-field modeling in solid-state phase transformation of steels was summarized by Lv et al. [20]. In situ SEM characterization and numerical modeling (using mean field and cellular automata models) of bainite formation and impingement of a medium-carbon, low-alloy steel was performed by Seppälä et al. [21].

Recently, some of the most effective methods for simulations of different phenomena and processes in materials science are the cellular automata (CA) method, the frontal cellular automata (FCA) method, and the lattice Boltzmann method (LBM). The LBM is based on microscopic particle models and mesoscopic kinetic equations and enables the modeling of complex multiphysics phenomena in a simple and flexible manner. Recently, Tong et al. [22] used the LBM in the simulation of coupled conduction and radiation heat transfer in composite materials, while Samanta et al. [23] presented the review of the application of the LBM for melting and solidification problems. CA, for example, was recently used for simulation of eutectic transformation during solidification of Al–Si alloys by Gu et al. [24], and for simulation of random diffusion of chloride in concrete under sustained load by Ma and Lin [25]. The FCA is one of the variants of CA, which allows one to speed up computations many times, and it can be used to model virtually all processes consisting of grain nucleation and growth. The FCA can be used to model different phenomena, for example, recrystallization [26], as well as manufacturing processes, for example, additive manufacturing [27] and rolling [28,29]. Considering that LBM comes from one of the CA variants known as LGA (lattice gas automata), it is possible to obtain a connection of FCA and LBM methods within a software interface, which can be characterized by high computational efficiency. The potential use of the CUDA parallel programming architecture further extends the possibilities to obtain effective and efficient numerical models.

The paper presents a three-dimensional model of heat flow during diffusional phase transformations with consideration of the enthalpy of transformation. This model can be considered as one of the main parts of a complex system for modeling diffusional phase transformations. The developed model is directly connected with the three-dimensional model of carbon diffusion, which is currently being developed. The 3D model is based on two modeling methods: FCA and LBM. The model takes into account the use of hybrid computing systems (CPU and GPU accelerators) for the calculations. Basic information on the FCA and LBM methods, as well as CUDA, which are the basis of the model, is shown. The paper briefly presents issues related to diffusional phase transformations, which can be modeled using the developed model. The scheme of the model and its main assumptions have been shown. Finally, examples of modeling results of austenite–ferrite transformation with a developed model are shown. NVIDIA graphics cards (GeForce RTX 2080 Ti, GeForce 1080Ti, GeForce 1060) were used for parallel calculations using the CUDA architecture. Open GL was used to visualize the results.

## 2. Diffusional Phase Transformations

Phase transformation processes in metals and alloys are one of the basic factors that can affect the structure and properties of materials. One of the basic phenomena that can affect the structure and properties of materials is phase transformations in metals and alloys. This area is very important in materials engineering because it allows, among others, the determination of the impact of changes in external conditions on the structure of metals and alloys. Obtaining the final product with appropriate properties is directly related to understanding the kinetics of transformations and elementary processes that occur in the material.

Diffusion processes are the basis of many metallurgical processes, such as diffusional phase transformations, formation of solid solutions (homogenization), recrystallization, thermochemical treatment, etc. Phase transformations are the basis for the heat treatment of alloys. They can be divided into two groups: diffusion and non-diffusion. Solid-state diffusional phase transformations represent a common phenomenon that when the temperature field and other external factors change in many alloys, the diffusion of components in grains and interfaces is activated, and then the migration of interfaces takes place. This process is accompanied by the nucleation, growth, and impingement of a new phase, changes in the volume fraction and chemical composition of the individual phases, and microstructure evolution [30]. Because the microstructure determines the material properties, it is of great theoretical and realistic importance to realize the accurate description of solid-state diffusional phase transformations and the precise control of the process to tailor the properties and optimize the hot working process of metals and alloys. Diffusionless transformations are also called martensitic. In steels, martensitic transformation can also be induced by cooling the austenite at a sufficiently high rate.

## 3. Frontal Cellular Automata and the Lattice Boltzmann Method

**Figure 2:**
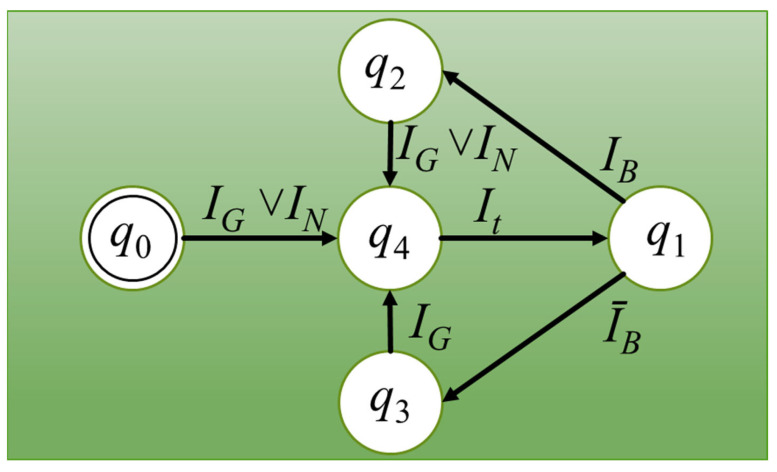
Universal FCA for microstructure studies.

The dimensions of the model space (in units of length) determine how large an area can be modeled. A regular grid is usually applied to this area. The number of elements is the basic parameter that influences the three main indicators of models based on cellular automata: resolution (accuracy), memory capacity, and calculation time. With a small amount of memory, the number of elements can be significantly limited, affecting the resolution or the size of the space. On the other hand, the use of a large memory (over 4–8 GB), i.e., a large number of cells, often leads to a significant increase in computation time, because it is at least linearly dependent on the number of elements. The simulation time depends on two factors: the number of time steps and the calculation time of one step. The number of steps often (but not always) depends directly on the linear dimension of the cell space (counted in cells). The computation time in one step usually depends linearly on the number of cells; i.e., in a three-dimensional space it is the cube of the cell space dimension (and in a two-dimensional space it is a square dependency). Then, the transition from a two-dimensional space of 1000 × 1000 cells to a three-dimensional space causes either a decrease in accuracy and resolution while maintaining the same number of cells (100 × 100 × 100), or a significant increase in the calculation time (about 3000 times) while maintaining the same linear size of the space (1000 × 1000 × 1000). The number of steps can be changed by changing the step length, and the calculation time can be changed in various ways. The first way can be described as extensive with the use of more computers, processors, processes, threads, or related to increasing the speed of processors, etc. The second can be called intensive and is directly related to the search for optimal methods and algorithms to solve the problem. The frontal cellular automaton is one of the variants of CA, which allows one to speed up the computation many times. The FCA scheme to study changes in the microstructure of materials is shown in Figure 2.

The basis of the FCA is the cell, which can be described by a number of parameter, such as, for example, location in space, states, and others. The description of the automaton consists of a set of states *Q*, the alphabet Σ, transition rules *δ*, and initial *Q_in_* and final states *Q_f_*. The description might look like this: *A* = {Q, Σ, *δ*, *Q_in_*, *Q_f_* }. The set of states *Q* = {*q*_0_, *q*_1_, *q*_2_, *q*_3_, *q*_4_} in the FCA was defined as follows: *q*_0_—initial state, *q*_1_—frontal state (cell at the front of changes), *q*_2_—cell at the grain boundary, *q*_3_—cell in the middle of the grain, and *q*_4_—transition state. The transient state is a state that introduces a delay to control the growth rate and is an active state like the frontal state. The states *q*_0_–*q*_3_ are essentially passive and are not taken directly into account in the calculations. In the initial microstructure algorithm, the input state may be *q*_0_. In other microstructure modeling algorithms, the input state may be *q*_2_ or *q*_3_. The final state is always formally *q*_2_ or *q*_3_, but in the case of an incomplete process, this state can be any. The cell alphabet contains “the letters” Σ = {*I*_G_, *I*_N_, *I*_t_, *I*_B_, ~*I*_B_} and is treated as a set of possible transition conditions between individual states, while the transition rules *δ* in the case of meeting the transition condition *I* (appearance of the appropriate letter) define the current state *q_i_* and the next state *q_i_*_+ 1_. A description of how the FCA works can be presented in a short form. The conditions *I_N_* (nucleation) and *I_G_* (grain growth) bring the cell into the *q*_4_ state, and at the same time the moment of transition is calculated. *I_t_* (the transition condition) changes the state of the cell to *q*_1_. *I_B_* (the condition “at the grain boundary”) determines whether the cell will be in the state *q*_2_ or *q*_3_. The proper definition of each of the basic conditions (*I_N_*, *I_G_*, and *I_t_*) is the essence of the adequate modeling of different microstructural phenomena and processes. An important feature of FCA is the closed circuit of transitions and states. Both the final and initial states can be repeated many times in subsequent modeling cycles, which is useful when simulating some phenomena and processes. Universal FCA can be used to model virtually all processes that consist of grain nucleation and growth. Details about the FCA can be found elsewhere [29].

The main idea of the LBM is to bridge the gap between the micro- and macroscale by not considering each particle’s behavior alone but rather the behavior of a collection of particles as a unit. The property of collecting particles is represented by a distribution function. The keyword is the distribution function. The distribution function acts as a representative of the collection of particles. This scale is called the meso-scale. LBM enjoys the advantages of both macroscopic and microscopic approaches with manageable computer resources. The basic LBM rule is the solution of the microscopic kinetics equation for the particle distribution function f(*x*,*u*,*t*), where (*x*,*u*) are the phase space variables and *t* is the time variable. The particle speed space ‘*u*’ can be effectively reduced to a set of discrete speeds {*e_i_* for *i* = 1,…,*b*} while keeping the hydrodynamic moments within the determined *u* range [31]. Such a discretization converts the Boltzmann equation into a discrete one taking the following form:

(1) $$f_{i}\left(x+e_{i},t+1\right)=f_{i}\left(x,t\right)-\frac{1}{\tau }\left[f_{i}\left(x,t\right)-f_{i}^{eq}\left(x,t\right)\right]+F_{i}\forall i=0,1,\ldots ,b$$

where *x*—dimensionless lattice units, {*e_i_*}—dimensionless set of discreet speeds, *τ*—relaxation time, $f_{i}^{eq}\left(x,t\right)=f_{i}^{eq}\left(\rho ,v\right)$—equilibrium distribution function of the *i*th discrete speed, *v—*macroscopic velocity, *ρ*—density, and *F_i_*—external forces (such as gravity). The numerical equation can be solved in two steps, namely transfer, propagation, or advection (streaming or advection step) and impact, collision, or relaxation (collision step), as follows:

(2) $$f_{i}^{\mathrm{in}}\left(x,t\right)=f_{i}^{\mathrm{out}\;}\left(x-e_{i},t-1\right)$$

(3) $$f_{i}^{\mathrm{out}\;}\left(x,t\right)=f_{i}^{\mathrm{in}\;}\left(x,t\right)-\frac{1}{\tau }\left[f_{i}^{\mathrm{in}\;}\left(x,t\right)-f_{i}^{eq}\left(x,t\right)\right]+F_{i}$$

During propagation, all distribution functions (except for *f*_0_) are transferred to the neighboring nodes of the grid according to their speed. Next, the particle distribution function approaches its equilibrium state due to collision. The input and output functions (that is, before and after the collision) are marked as “in” and “out”. The macroscopic density *ρ* and moment *ρv* are the first and the second moments of the distribution function, as follows:

(4) $$\rho =\overset{b}{\underset{i=0}{\sum }}f_{i}$$

(5) $$\rho v=\overset{b}{\underset{i=0}{\sum }}f_{i}e_{i}$$

The viscosity is derived from the following equation: $v=\frac{1}{3}\left(\tau -\frac{1}{2}\right)$. Distribution function change equations for diffusion, convection, and heat transfer are as follows:

(6) $$g_{i}\left(x+e_{i},t\right)=g_{i}\left(x,t\right)-\frac{1}{\tau_{T}}\left[g_{i}\left(x,t\right)-g_{i}^{eq}\left(x,t\right)\right]+\frac{1}{b_{T}}Q\;\;\;\;\;\forall i=1,\ldots ,b_{T}$$

where *τ_T_*—relaxation time for heat transfer (diffusion), and *Q*—heat source (elements). The equilibrium function, for example, for the D2Q4 model is calculated in the following way:

(7) $$g_{i}^{eq}\left(x,t\right)=\frac{1}{4}T\left[1+2e_{i}\cdot v\right]\;\;\forall i=1,\ldots ,4$$

The temperature may be computed using the following equation:

(8) $$T=\overset{b_{T}}{\underset{i=0}{\sum }}g_{i}$$

**Figure 3:**
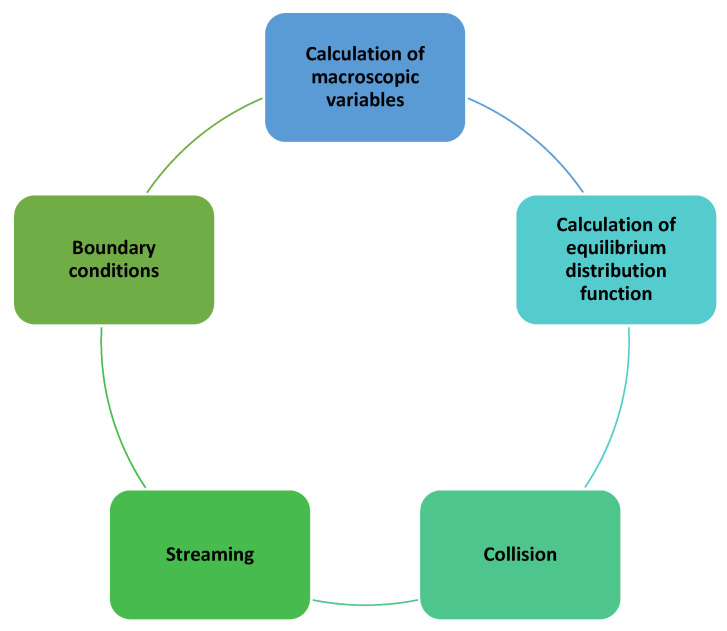
The general LBM calculation scheme.

Modeling using LBM is a cyclical repetition of several steps or operations (Figure 3).

A detailed description of LBM can be found elsewhere [32].

## 4. CUDA Parallel Computing Platform

Taking into account some features of the LBM method, this method was not widely used to model different phenomena and processes. The main disadvantages were the high memory requirements and the large number of iterations related to the calculation time. The development of computer and computational techniques related to the possibility of using parallel calculations (GPU accelerators) changed the perception of this method from not very useful to very effective.

Modern computer systems are characterized by parallelism. Programming techniques suitable for a traditional single-core processor do not provide the ability to use the computing power of a device with even only 100 computing cores. As a result, many different solutions have been created that support parallel programming. The CUDA (compute unified device architecture) technology developed by NVIDIA has gained great popularity in the field of universal calculations performed with the help of graphics cards. The standard solution in this area is OpenCL, and the undoubted advantage of which is that it works with graphics cards from different manufacturers. It can also be used for other technological solutions than graphics cards [33].

The parallel solution of the problem requires dividing the set into smaller parts. In general, there are two ways to divide a given task: the first is “divide and conquer” and the second is “scatter and take”. In the first method, a given problem is broken down into smaller problems that are easier to solve. The right combination of solutions to smaller problems allows researchers to solve the main problem. The second approach is similar, but instead of dividing the whole problem into smaller subproblems, researchers just separate the data and pass those chunks to the computational units. The subsequent collection of data and their additional ordering allows for the correct completion of the calculations. In both cases, however, the problem or the data are broken down into smaller parts. Therefore, an important aspect of parallel calculations is data independence. Very good performance can be obtained quite simply if the data can be easily divided into independent groups. In the case of parallel programming, data synchronization mechanisms are needed both at the level of individual threads or work units, as well as between individual stages of the entire algorithm.

CUDA is nowadays the leading technology for general-purpose computations on GPUs. The great popularity of CUDA technology results from its far-reaching integration with the C and C++ languages. The use of special features of the CUDA architecture is made possible by extending the C and C++ languages with a number of additional keywords to support this architecture. CUDA C became the first programming language developed by a GPU manufacturing company for general computing. NVIDIA also provides a special hardware driver that allows one to take advantage of the powerful computing power of the CUDA architecture. Solutions built on the basis of NVIDIA graphics processors enjoy a better ratio of performance to price and performance to the amount of power consumed compared to traditional solutions.

**Figure 4:**
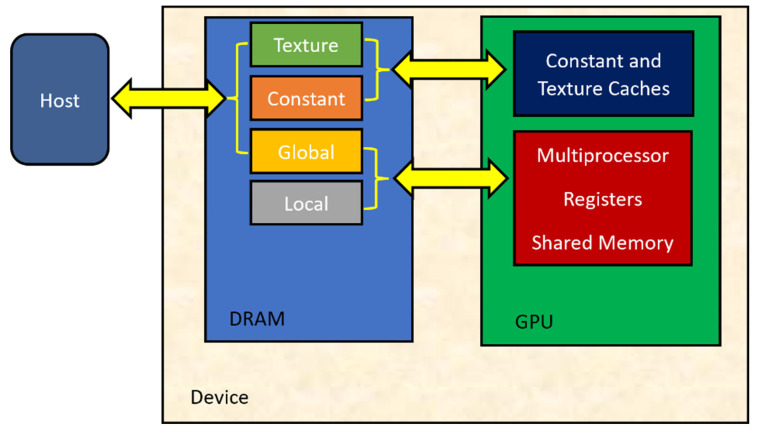
CUDA memory spaces.

CUDA defines both a programming model and general hardware specifications. The CUDA architecture uses one combined processing pipeline. Due to this, the program performing general calculations has all the arithmetic logic units (ALUs) of the processor at its disposal. The ALUs are built according to the IEEE standard for single-precision floating-point arithmetic and have a built-in set of instructions that are dedicated to general computing rather than graphics processing. GPU execution units also have random access to memory for reading and writing, as well as to a software-managed cache, called shared memory. Memory spaces are shown in Figure 4. Global, local, and texture memory has the highest access latency, followed by constant memory, shared memory, and the register file [34].

**Figure 5:**
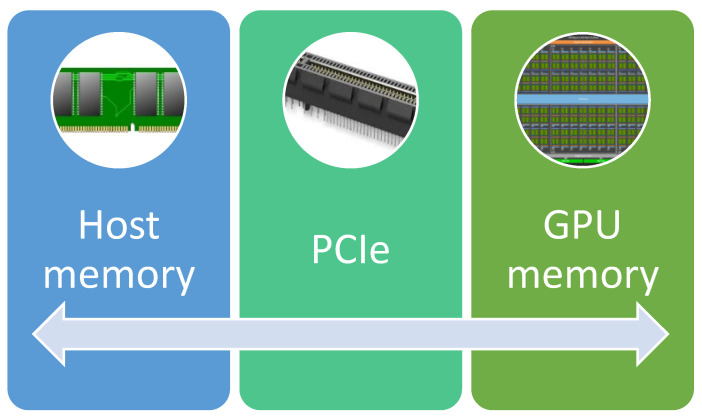
Connection between host and GPU memory.

GPUs are usually connected to the motherboard over a PCIe connection, and data from the main (host) memory must be transferred to the GPU memory over this PCIe link. Figure 5 shows the PCIe connection.

CUDA-capable GPUs consist of a set of streaming multiprocessors (SM), each containing several scalar processors (SP). A CUDA program basically consists of CPU code and (at least) one kernel, i.e., a void-returning function to be executed by the GPU. Kernels are executed in several threads with private local variables. Threads are grouped into identical blocks that may have up to three dimensions. During execution, a block cannot be partitioned and, therefore, must fit into a single SM. However, an SM may execute several blocks simultaneously. Threads within a block may be synchronized and have access to a shared memory space.

## 5. 3D Heat Flow Model

**Figure 6:**
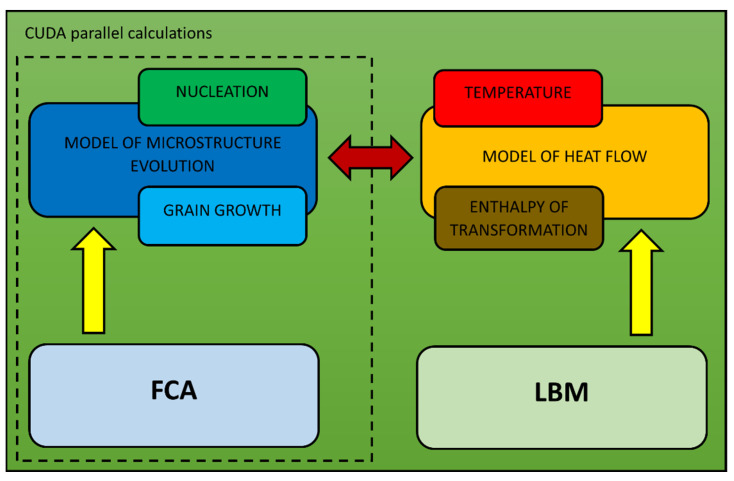
3D heat flow model based on LBM and the connection with the microstructure evolution model.

The 3D heat flow model developed on LBM, which can be used to model heat transfer during diffusional phase transformations, is shown in Figure 6. The main parameters considered in the model are temperature and enthalpy of transformation. This model is directly connected to the microstructure evolution model, which is based on the FCA method. In the model, the CUDA parallel calculations are used.

**Figure 7:**
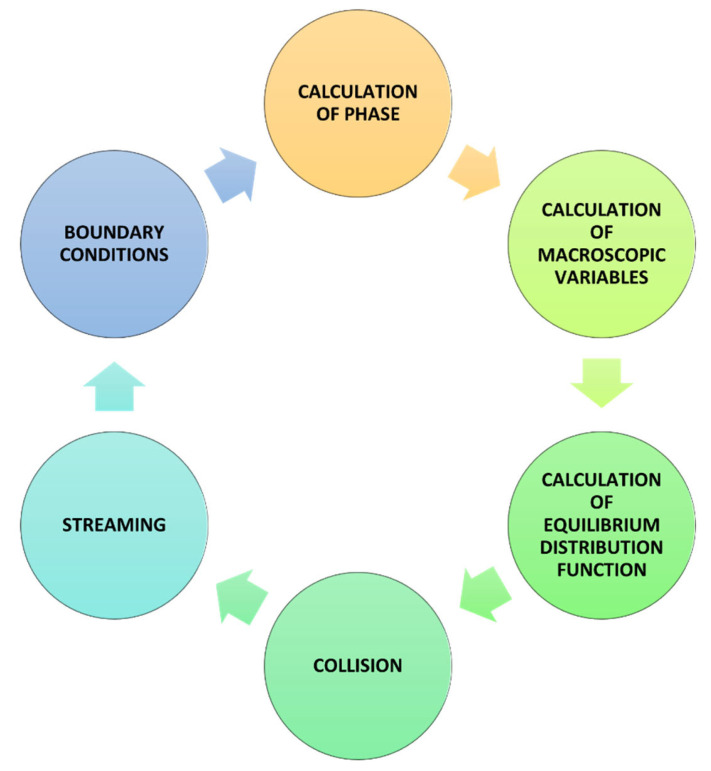
Phase calculation in the LBM algorithm.

The calculations of heat flow during phase transformations are based on the general LBM modeling scheme with an extension of an additional element related to the phase calculation (Figure 7).

The calculations in the model are based on the 3D Fourier equation, which describes the heat transfer as follows:

(9) $$\frac{1}{a}\frac{\partial T}{\partial t}=\left(\frac{\partial^{2}T}{\partial x^{2}}+\frac{\partial^{2}T}{\partial y^{2}}+\frac{\partial^{2}T}{\partial z^{2}}\right)+Q\left(x,y,z,t\right)$$

where *T*—temperature, *a*—thermal diffusivity, and *Q*(*x*,*y*,*z*,*t*)—the source of thermal energy.

The Bhatnagar–Gross–Krook (BGK) collision model is one of the models that can be used in LBM simulations. The model approximates the collision term of the Boltzmann equation by a single relaxation process from a non-equilibrium state to an equilibrium state and was used for modeling of heat flow. A simple discretization of the Boltzmann equation with BGK operator can be presented by the following expression:

(10) $$\begin{array}{c} f_{k}\left(x+\Delta x,y+\Delta y,z+\Delta z,t+\Delta t\right)-f_{k}\left(x,y,z,t\right)=\\ -\frac{\Delta t}{\tau }\left[f_{k}\left(x,y,z,t\right)-f_{k}^{eq}\left(x,y,z,t\right)\right]+\Delta tw_{k}S \end{array}$$

where $f_{k}\left(x,y,z,t\right)$ and $f_{k}^{eq}\left(x,y,z,t\right)$ are the particle and equilibrium distribution functions, respectively, *τ* is the single-relaxation-time parameter, *w_k_* stands for the weighting factor in direction *k*, and *S* is the source term.

The equilibrium distribution function can be calculated as follows:

(11) $$f_{k}^{eq}=w_{k}T\left(x,y,z,t\right)$$

where *T* is the temperature.

Collision and streaming are two important steps in the LBM algorithm, and they can be described by the following expressions (according to Equations (2) and (3)).

Collision is depicted as follows:

(12) $$f_{i}\left(x,y,z,t+\Delta t\right)=f_{k}\left(x,y,z,t\right)\left[1-\omega \right]+\omega f_{k}^{eq}\left(x,y,z,t\right)$$

where *ω* = Δ*t*/*τ*.

Streaming is depicted as follows:

(13) $$f_{i}\left(x+\Delta x,y+\Delta y,z+\Delta z,t+\Delta t\right)=f_{k}\left(x,y,z,t+\Delta t\right)$$

**Figure 8:**
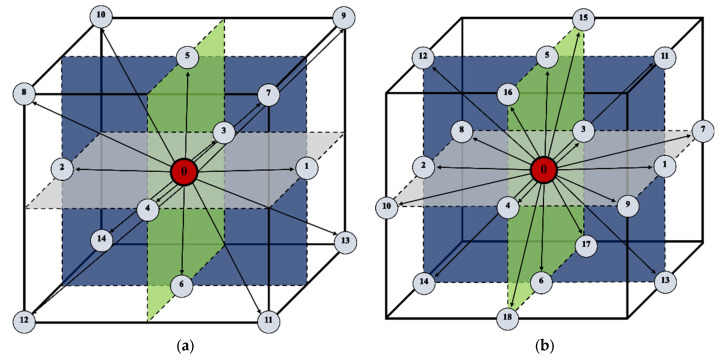
D3Q15 (**a**) and D3Q19 (**b**) LBM lattices.

Figure 8 shows the D3Q15 and D3Q19 schemes, which were used for the simulations. The lattice velocities *e_i_* and the corresponding lattice factors for the calculation schemes are as follows:

- D3Q15: *e*_(0)_ = (0, 0, 0), *e*_(1,2)_ = (±1, 0, 0), *e*_(3,4)_ = (0, ±1, 0), *e*_(5,6)_ = (0, 0, ±1), *e*_(7–14)_ = (±1, ±1, ±1), and *w*_(0)_ = 2/9, *w*_(1–6)_ = 1/9, *w*_(7–14)_ = 1/72.
- D3Q19: *e*_(0)_ = (0,0,0), *e*_(1,2)_ = (±1, 0, 0), *e*_(3,4)_ = (0, ±1, 0), *e*_(5,6)_ = (0, 0, ±1), *e*_(15,16,17,18)_ = (±1, ±1, 0), *e*_(11,12,13,14)_ = (±1, 0, ±1), *e*_(7,8,9,10)_ = (0, ±1, ±1), and *w*_(0)_ = 1/3, *w*_(1–6)_ = 1/18, *w*_(7–18)_ = 1/36.

**Figure 9:**
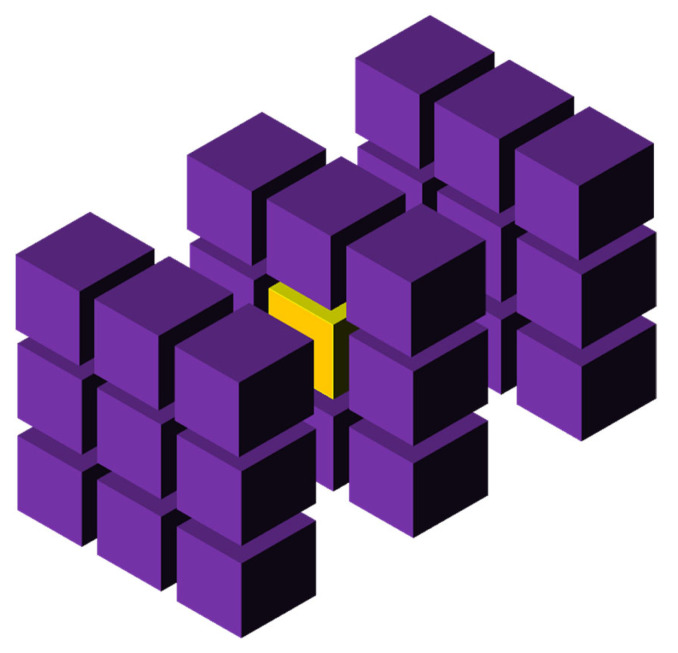
3D Moore neighborhood.

One of the most important parameters defined in cellular automata is neighborhood. Two kinds of cell neighborhood, namely von Neumann’s and Moore’s, are usually considered for space. The simulations were performed using the three-dimensional Moore neighborhood (Figure 9).

The reconstruction of the real surface (boundary) based on the values of the volume fractions of several cells (volume fraction refers to a specific new phase in the cell) often required the introduction of the vector normal to the surface. The 3D heat flow model includes one of the commonly used methods for calculating the normal vector. This method is based on the calculation of the center of mass [35].

**Figure 10:**
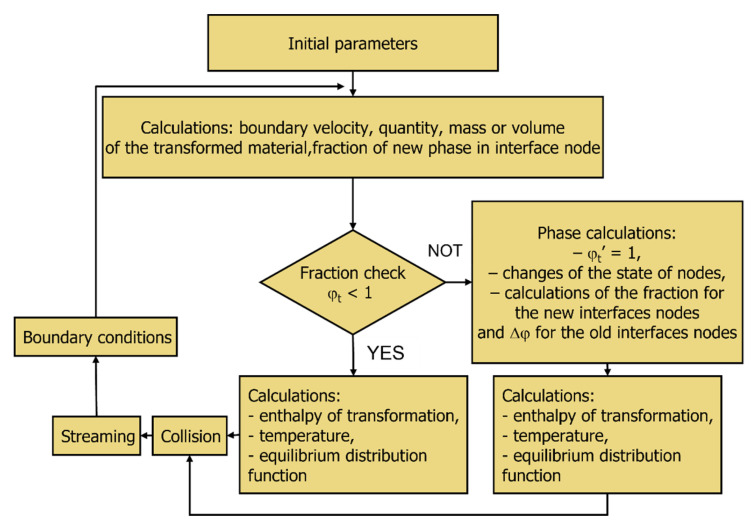
Algorithm for 3D simulation of heat flow during phase transformation.

Figure 10 shows the LBM numerical algorithm, which is used for 3D modeling of the heat flow during phase transformation considering the enthalpy of the transformation.

The calculation algorithm includes the following repeated steps (steps 2–9 are repeated in the cycle):

1. Determination of the initial simulation and model parameters:
  - Number of nodes: *nx*, *ny* and *nz*;
  - Number of time steps: *tsteps*;
  - Position of the node: *x*, *y*, *z*;
  - Grid step: Δ*x*, Δ*y*, Δ*z*;
  - Time step: Δ*t*;
  - Fractions of phases austenite (γ) and ferrite (α) in interface nodes: *ϕ_I__γ_* = 1 and *ϕ_I__α_* = 0;
  - Thermal diffusivity: *a*;
  - Velocity coefficient: *k_v_*;
  - Specific enthalpy coefficient: *k_q_*;
  - Temperature of phase transformation: *T_P_*;
  - Interface temperature: *T_I_*;
  - Node temperature: *T_x,y,z_*.
2. Calculation of a boundary velocity *v*: *v* = *k_v_(T_P_*-*T_I_*)
3. Determination of the quantity, mass, or volume of the transformed material in interface node: *Δϕ_I_* = *vΔt*.
4. Changes in the new phase fraction: *ϕ_I__α__,t_* = *ϕ_I__α__,t_*_-1_ + Δ*ϕ_I_*
5. Fraction checking:
  (a) if *ϕ_Iα,t_* < 1 ⇒ go to point 6;
  (b) if *ϕ_Iα,t_* ≥ 1 ⇒ *ϕ_I__α__,t_*’ = 1, change the state of the node from I to α and the neighboring nodes from γ—austenite to I—interface (3D Moore neighborhood), set the fraction value for the new I: Δ*ϕ_nI_* = (*ϕ_Iα,t_*—*ϕ_I__α__,t_*’)/*numγ* = *ϕ_I__α__,nI_*; *numγ*—the number of nodes in phase γ in the neighborhood of old I, the Δ*ϕ* for the old I (new α—ferrite) node: Δ*ϕ_oI_* = 1—ϕ*_I__α_*_,*t*-1_; *ϕ_I__α__, oI_* = 1; *ϕ_I__γ__, oI_* = 0.
6. Calculation of the heat source during the transformation:
  (a) if *ϕ_t_* < 1 ⇒ *Q_I_* = *k_q_*Δ*ϕ_I_*;
  (b) if ϕ*_t_* ≥ 1 ⇒ *Q_nI_* = *k_q_*Δϕ*_nI_; Q_oI_* = *k_q_*Δϕ*_oI_*;
  (c) calculation of the new temperature in the nodes: *T_x,y,z_ = Σf_i_ + Q_x,y,z_*’
  (d) *f^eq^* calculation.
7. Calculation of *f^out^*—collision.
8. Streaming.
9. Application of boundary conditions.

The parameters calculated in steps 2–7 are considered as the output parameters of the simulations.

## 6. Simulation Results

The 3D model of heat flow, which takes into account the enthalpy of transformation, was multidimensionally analyzed, and the influence of the main parameters considered in the model on heat transfer was modeled. The thermal diffusivities *a,* the specific enthalpy coefficient *k_q_*, and the velocity coefficient *k_v_* are the main parameters considered in the heat flow model. The basic connection obtained with the microstructure evolution model based on the FCA method also allowed observation of the influence of the main parameters included in the heat flow model, for example, on the rate of transformation. The thermal diffusivities *a,* the specific enthalpy coefficient *k_q_*, and the velocity coefficient *k_v_* are the main parameters considered in the heat flow model. The selected results of the three-dimensional heat transfer modeling are presented for the selected values of the parameters defined in the model as well as the changes in the microstructure.

**Figure 11:**
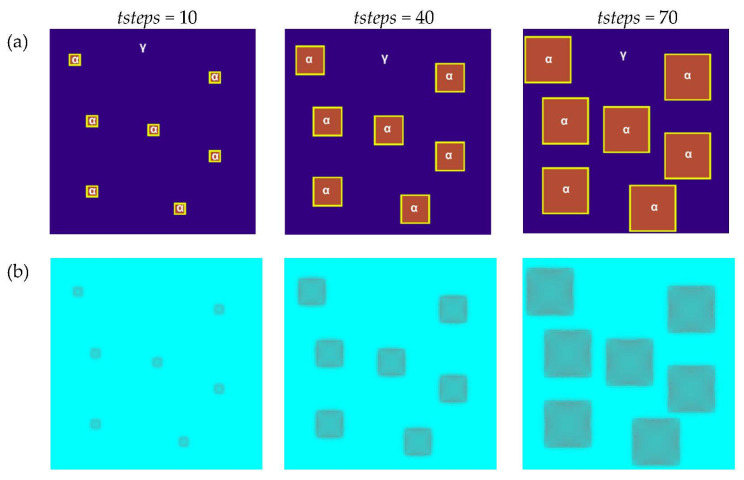
Grain growth of the new phase with the use of the Moore neighborhood at various stages of growth: (**a**) FCA states, and (**b**) temperature distributions.

The first of the presented modeling variants concerns the modeling of heat flow in a system composed of seven randomly distributed grains and the use of Moore’s neighborhood. The growth of the grains and changes in temperature were modeled. The second modeling variant shows the use of a normal vector to the surface, which was introduced for the calculations. The enthalpy of transformation was taken into account. The simulations were carried out using the D3Q19 LBM scheme (Figure 8b). The basic parameters used for the modeling are as follows: number of nodes—*n_x_* × *n_y_* × *n_z_* = 120 × 120 × 120, Δ*x* = 1, Δ*y* = 1, Δ*z* = 1, Δ*t* = 1, *τ* = 1, *k_v_* = 0.004, and *k_q_* = 15. The bounce-back boundary conditions were applied at the edges. The temperature of phase transformation was equal to 810 °C, while the initial temperature for all nodes was set to 760 °C. The calculations were performed on NVIDIA GeForce GTX 1060 and NVIDIA GeForce GTX 2080Ti graphics cards using the CUDA parallel programming architecture. Figure 11a shows the grains’ growth with the use of the Moore neighborhood at various stages of growth for one of the selected planes (*x* =1, 2, …, *n_x_*, y = *n_y_*/2, *z* = 1, 2, …, *n_z_*) in the 3D model space. The temperature distributions are shown in Figure 11b. The blue color represents 760 °C.

**Figure 12:**
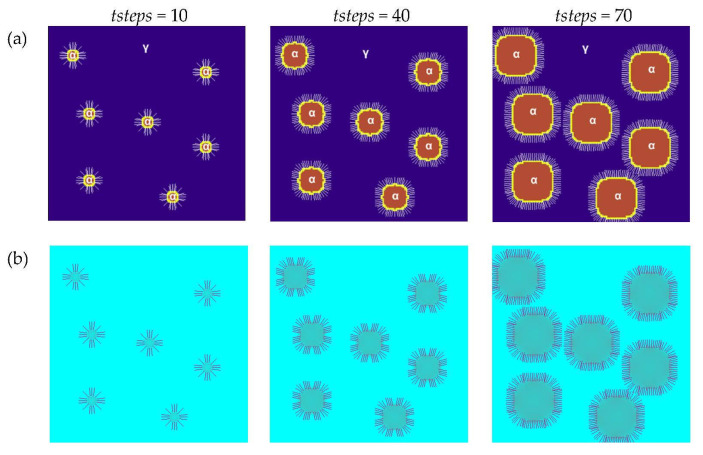
Grain growth of the new phase with the use of the Moore neighborhood and vector normal to the surface in various stages of growth: (**a**) FCA states and (**b**) temperature distributions.

The second modeling variant shows the use of a normal vector to the surface, which was introduced for the calculations. The other simulation parameters were the same as in the first variant. Figure 12a shows the growth of the grains in various stages of growth for the same selected plane as in the first variant using the Moore neighborhood and the vector normal to the surface. The introduction of such a vector was based on the calculation of the center of mass and directly influences the calculation of Δ*ϕ_I_*. The temperature distributions are presented in Figure 12b. The introduction of a normal vector reduces anisotropy. The normal vectors to the surface are shown by the white lines (Figure 12a). Different positions of the vectors can be observed, and this affects the growing conditions.

The next presented modeling results refer to the influence of parameters *a*, *k_q_*, and *k_v_* on the heat flow during the transformation and grains growth. The research was carried out for another of the selected planes (*x* = *n_x_*/2, y = 1, 2, …, *n_y_*, *z* = 1, 2, …, *n_z_*) in the 3D modelled space. The structure with five randomly distributed grains was analyzed. The basic parameters used for the modeling were as follows: a number of nodes—*n_x_* × *n_y_* × *n_z_* = 120 × 120 × 120, *tsteps* = 50, Δ*x* = 1, Δ*y* = 1, Δ*z* = 1, Δ*t* = 1, bounce-back boundary conditions. The initial temperatures at the nodes and the temperature of phase transformation were the same as in the first two variants previously presented.

**Figure 13:**
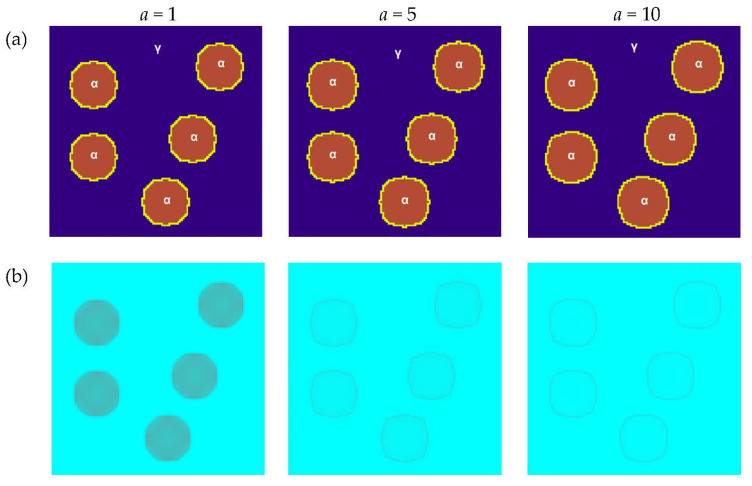
Growth of grains of the new phase with the use of the Moore neighborhood and a vector normal to the surface for different values of the thermal diffusivity coefficient: (**a**) FCA states and (**b**) temperature distributions.

The study of thermal diffusivity influence was carried out for constant values of *k_q_* = 15 and *k_v_* = 0.006. Figure 13 shows the grain growth and temperature distribution at different stages for different values of the *a* coefficient. A small value of thermal diffusivity means that the heat is mostly absorbed by the material and a small amount of heat is conducted further. For its greater value, the heat conduction is faster and, because the temperature governs the speed of chemical reactions, this will influence the rate of transformation. For a smaller value of *a,* the heat transfer is slower. This relationship can be observed in the results obtained and is consistent with the available data in the literature [36].

**Figure 14:**
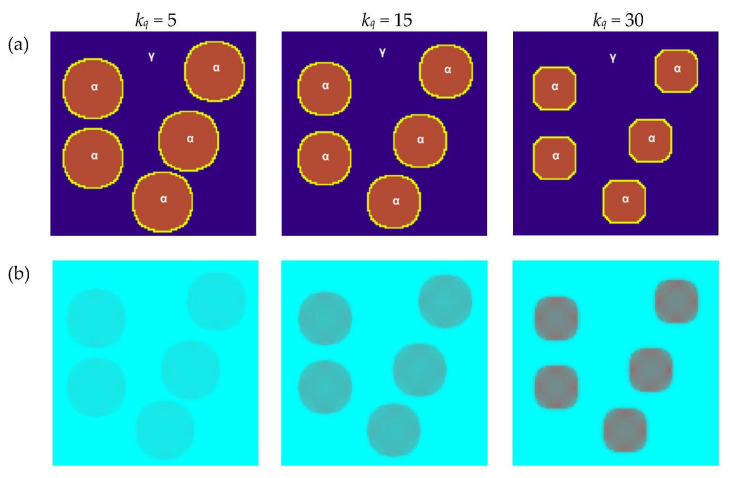
Growth of grains of the new phase with the use of the Moore neighborhood and the normal vector to the surface for different values of the specific enthalpy coefficient: (**a**) FCA states and (**b**) temperature distributions.

Another of the parameters studied was the specific enthalpy coefficient *k_q_*. Changes in microstructure and temperature distribution for different values of the *k_q_* parameter were simulated by assuming the same values of the base parameters as in the previous variant, where the influence of *a* was studied. The same plane was selected, and constant values of *tsteps* = 70, *a* = 1 and *k_v_* = 0.005 were established. The results are presented in Figure 14. Specific enthalpy generally refers to the enthalpy per unit mass. The specific enthalpy coefficient relates the quantity of the transformed material in the interface node (Δ*ϕ*_I_) with the heat generated or absorbed during the transformation of such a quantity of matter (*Q*). For lower values of *k_q_*, the rate of phase transformation is higher, which is related to the fact that the temperature in the interface grows slower and this has an impact on higher values of boundary velocity. The aspect of transformation enthalpy is considered in many works [37] and the results obtained in the context of the impact on transformation are comparable with them.

**Figure 15:**
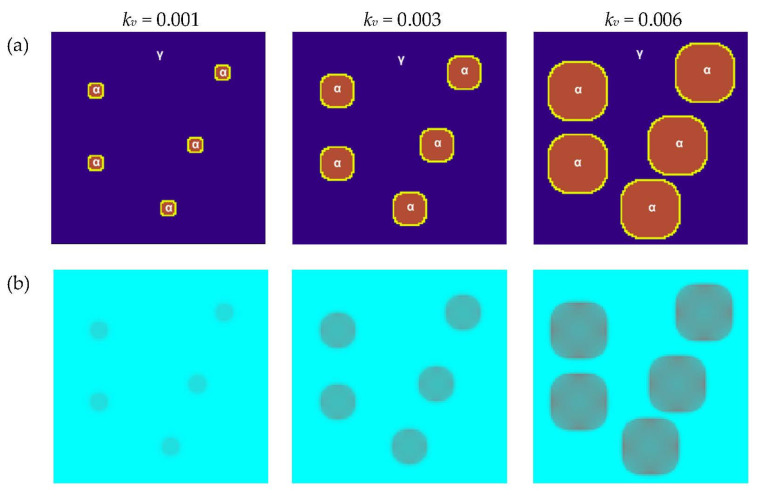
Growth of grains of the new phase with the use of the Moore neighborhood and the vector normal to the surface for different values of velocity coefficient: (**a**) FCA states and (**b**) temperature distributions.

The influence of the *k_v_* coefficient is shown in the last variant of the simulation presented (Figure 15). The velocity coefficient is a design factor that relates the overheating/overcooling (Δ*T*) during the transformation with the boundary velocity (*v*). Each transformation for the given overheating/overcooling has its own values of velocity coefficient. The *k_v_* is used for the calculation of the velocity, which determines the amount of transformed matter, and this coefficient also influences the rate of transformation. The basic parameters for the simulations in this variant did not change in relation to the previous two variants where the influence of *a* and *k_q_* were studied. Additionally, *tsteps* = 70, *a* = 1, and *k_q_* = 20 were assumed. The results obtained can be directly related to the data presented in the literature [38].

Every transition rule of CA based on the short-range neighborhood (von Neumann, Moore, hexagonal, etc.) introduces anisotropy into the CA model connected with the privileged directions of information spread. If the rules are based only on the neighborhood, they determine the shape of the growing grains. The Moore neighborhood in 2D imposes a shape of a square with the sides oriented along the coordinate directions and a cubic shape in 3D. When grain growth depends additionally on the gradient of elements concentration and consequently on diffusion, homogeneity (or isotropy) of grain growth is reduced. It can be explained that diffusion is 3D near the top of the grain, 2D near the edge, and 1D near the surface. Therefore, unnatural space anisotropy is straightened by natural diffusion. To approach real processes, the anisotropy of modeled space should be eliminated or at least reduced. A normal vector is introduced into the model for this aim and helps maintain the initial shape of the grains. The homogeneity of grain growth depends on the grain shape and condition of the diffusion. Taking into account the presented results, it can be seen that the new phase grows due to the movement of phase interface, while the coefficients *a*, *k_v_*, and *k_q_* determine the velocity of the interface movement. As the temperature in the interface increases, the heat is transferred to the neighboring nodes and has an influence on their temperature. The temperature change at the interface also influences the boundary velocity.

## 7. Summary

By using technological processes that change the structure of the material, its properties can be deliberately shaped within certain limits. A structure’s quality is clearly dependence upon mechanical, electrical, and magnetic properties and corrosion resistance. Phase transformations are very important from the point of view of shaping the appropriate properties of materials, including those taking place in carbon steels, called diffusion phase transformations. Numerical modeling still plays an important role in this area, and the newly developed models use different approaches and different methods to model the formation of the microstructure after transformation. This paper presents the 3D heat flow model for modeling of heat transfer during diffusional phase transformations, which is based on the LBM method. This model takes into account the enthalpy of transformation. The connection was obtained with the microstructure evolution model based on the FCA method. The calculation algorithm for the heat flow is presented in detail, taking into account the enthalpy of the transformation. Examples of simulation results of heat flow and growth of the new phase in the selected planes of the 3D modeling space for different simulation conditions are also presented. The D3Q19 LBM scheme and the Moore neighborhood were used for the simulations. The vector normal to the surface was also introduced for the calculations in the next stage of model development. The heat flow model also uses CUDA parallel computing on GPUs. A basic link was obtained between the developed heat flow model and the microstructure evolution model. This is an important step in obtaining the comprehensive heat flow and carbon diffusion model, which can be used for comprehensive modeling of different diffusional phase transformations. The developed model is limited to modeling only one type of transformation. The size and resolution of the modeled space are limited by parameters of specific graphics cards for calculations.

Potential directions for future investigations are as follows:

- Implementation of carbon diffusion in the steel into the developed 3D model.
- Implementation of 3D models of heat flow and carbon diffusion into the comprehensive 3D model of microstructure evolution.
- Extending the modeling capabilities to a different type of transformation.

## Data Availability

Not applicable.

## Nomenclature

*a*	thermal diffusivity
D3Q15, D3Q19	LBM schemes
{*e_i_*}	set of discrete speeds
*F_i_*	external forces
$f_{k}\left(x,y,z,t\right)$	particle distribution function
$f_{k}^{eq}\left(x,y,z,t\right)$	equilibrium distribution function
*I_N_*	nucleation condition
*I_G_*	grain growth condition
*I_t_*	transition condition
*I_B_*	condition “at the grain boundary”
*k_v_*	velocity coefficient
*k_q_*	specific enthalpy coefficient
*nx*, *ny*, *nz*	number of nodes
*q*_0_	initial state
*q*_1_	frontal state
*q*_2_	cell at the grain boundary
*q*_3_	cell in the middle of the grain
*q*_4_	transition state
*Q*	heat source
*Q* = {*q*_0_, *q*_1_, *q*_2_, *q*_3_, *q*_4_}	set of frontal cellular automata states
*Q*(*x*,*y*,*z*,*t*)	the source of thermal energy
*S*	source term
*tsteps*	number of time steps
Δ*t*	time step
*T*	temperature
*T_I_*	interface temperature
*T_P_*	temperature of phase transformation
*T_x,y,z_*	node temperature
*v*	boundary velocity
*w_k_*	weighting factor in direction k
*x*, *y*, *z*	position of the node
Δ*x*, Δ*y*, Δ*z*	grid step
Σ	alphabet
*δ*	transition rules
*ρ*	density
*ρv*	moment
*τ*	relaxation time
*τ_T_*	relaxation time for heat transfer (diffusion)
Δ*ϕ_I_*	quantity, mass, or volume of the transformed material in interface node
*ϕ_Iα_*	fractions of ferrite (α) in interface node
*ϕ_Iγ_*	fractions of austenite (γ) in interface node
*ω*	collision frequency

Abbreviations

CA	cellular automata
CUDA	compute unified device architecture
FCA	frontal cellular automata
LBM	lattice Boltzmann method
LGA	lattice gas automata
